# Decoding Bilateral Hindlimb Kinematics From Cat Spinal Signals Using Three-Dimensional Convolutional Neural Network

**DOI:** 10.3389/fnins.2022.801818

**Published:** 2022-03-25

**Authors:** Yaser Fathi, Abbas Erfanian

**Affiliations:** ^1^Department of Biomedical Engineering, School of Electrical Engineering, Iran Neural Technology Research Centre, Iran University of Science and Technology, Tehran, Iran; ^2^School of Cognitive Sciences, Institute for Research in Fundamental Sciences (IPM), Tehran, Iran

**Keywords:** neural decoding, spinal cord, locomotion, descending tracts, ascending tracts, convolutional neural network

## Abstract

To date, decoding limb kinematic information mostly relies on neural signals recorded from the peripheral nerve, dorsal root ganglia (DRG), ventral roots, spinal cord gray matter, and the sensorimotor cortex. In the current study, we demonstrated that the neural signals recorded from the lateral and dorsal columns within the spinal cord have the potential to decode hindlimb kinematics during locomotion. Experiments were conducted using intact cats. The cats were trained to walk on a moving belt in a hindlimb-only condition, while their forelimbs were kept on the front body of the treadmill. The bilateral hindlimb joint angles were decoded using local field potential signals recorded using a microelectrode array implanted in the dorsal and lateral columns of both the left and right sides of the cat spinal cord. The results show that contralateral hindlimb kinematics can be decoded as accurately as ipsilateral kinematics. Interestingly, hindlimb kinematics of both legs can be accurately decoded from the lateral columns within one side of the spinal cord during hindlimb-only locomotion. The results indicated that there was no significant difference between the decoding performances obtained using neural signals recorded from the dorsal and lateral columns. The results of the time-frequency analysis show that event-related synchronization (ERS) and event-related desynchronization (ERD) patterns in all frequency bands could reveal the dynamics of the neural signals during movement. The onset and offset of the movement can be clearly identified by the ERD/ERS patterns. The results of the mutual information (MI) analysis showed that the theta frequency band contained significantly more limb kinematics information than the other frequency bands. Moreover, the theta power increased with a higher locomotion speed.

## Introduction

Neural interfaces have the potential to restore limb function by translating neural activities into command signals that could be used in a neuroprosthetic device for people with spinal cord injury (SCI) (Jackson and Zimmermann, 2012). Restoring the lost function, such as locomotion, requires the integration of both motor and sensory information. Sensory information is an essential requirement for the closed-loop control of locomotion. Many attempts have been made to obtain sensory information from different parts of the nervous system, such as the peripheral nerve (Riso et al., 2000; Micera et al., 2001; Hansen et al., 2004; Song et al., 2017), dorsal root ganglia (DRG) (Stein et al., 2004; Weber et al., 2006, 2007; Rigosa et al., 2011; Wagenaar et al., 2011; Bruns et al., 2013; Holinski et al., 2013; Han et al., 2017; Kashkoush et al., 2019), ventral roots (Debnath et al., 2014), and spinal cord gray matter (Yeganegi et al., 2018; Fathi and Erfanian, 2019). Moreover, intracortical recordings were used to decode the hindlimb kinematics and muscle electromyographs (EMGs) during bipedal walking in monkeys (Fitzsimmons et al., 2009) and rats (Rigosa et al., 2015) and during quadrupedal locomotion in rats (Song et al., 2009; Barroso et al., 2019). These studies suggest that cortical recording can be used to bypass spinal cord injuries. Several studies have examined the intracortical regulation of the spinal circuit through epidural electrical stimulation (Capogrosso et al., 2016; Knudsen and Moxon, 2017; Bonizzato et al., 2018).

Another possible source of hindlimb kinematic information is the descending pathways (i.e., motor tracts). Motor signals are sent from the brain to the spinal cord through descending tracts. The main descending pathways, including the corticospinal and rubrospinal tracts, play a significant role in controlling balance, posture, locomotion, and reaching. Reticulospinal pathways are also critical in initiating locomotion, postural control, and postural muscle tone. The rubrospinal and corticospinal tracts together with the medullary reticulospinal tract project all to the lateral columns in spinal cord (Lemon, 2008; Rossignol et al., 2009; Frigon et al., 2011; Takakusaki et al., 2016). The previous studies revealed that there was a correlation between the neural signals recorded from the descending tracts and forelimb movements (Prasad and Sahin, 2006, 2010, 2012; Guo et al., 2014). Recently, it was demonstrated that the EMG signals of the forelimb flexor and extensor can be decoded from the lateral columns (Guo et al., 2018; Gok and Sahin, 2019).

In the previous study, we decoded the kinematics of only one leg using the neural signals recorded from the lateral and dorsal columns on one side of the spinal cord during cat hindlimb-only locomotion (Fathi and Erfanian, 2021). A linear regression model was used for decoding hindlimb joint angles. In the current study, we expanded the previous work and decoded the kinematics of both hindlimbs from neural activity recorded from the descending lateral tracts and from the ascending tracts during voluntary hindlimb-only walking on the treadmill. Moreover, we demonstrated that the neural signals recorded from the spinal cord could reflect the speed of locomotion.

Another critical issue in decoding kinematic information from neural signals is the decoding model. Different decoding methods have been employed for this purpose. Linear regression methods were used to estimate the hindlimb kinematics from populations of neurons in the DRG (Stein et al., 2004; Weber et al., 2006, 2007; Bruns et al., 2013; Holinski et al., 2013). To improve the performance of decoding limb kinematics, different nonlinear methods, including particle filtering (Wagenaar et al., 2011), fuzzy neural network (FNN) model (Rigosa et al., 2011), and recurrent neural networks (RNNs) (Han et al., 2017; Yeganegi et al., 2018) have also been employed. It was demonstrated that the nonlinear model provided better decoding accuracy than the multiple linear regression and Kalman filter estimators (Han et al., 2017). Recently, we proposed a probabilistic recurrent neural network (PRNN) for decoding hindlimb kinematics from neural activity recorded from the dorsal horn of the spinal cord. We demonstrated the superiority of the PRNN over the conventional recurrent neural network and Kalman filter (Fathi and Erfanian, 2019).

In contrast to the previous work, in which a linear regression model was used for decoding, in the current study, a three-dimensional convolutional neural network (3D-CNN) was employed for decoding kinematic information. In recent years, tremendous interest in CNNs has emerged as the most established deep learning algorithm. CNN is a biologically inspired model that can learn high-level local features by convolving a set of filters with input data (LeCun et al., 2015; Patterson and Gibson, 2017). CNNs have achieved significant progress in image processing (Li et al., 2021), video (Liu et al., 2020), speech (Mustaqeem and Kwon, 2020), and natural language processing (Widiastuti, 2019; Li et al., 2020). CNNs have also been applied extensively in cortical signal processing for different applications (Zhang et al., 2021) such as brain-computer interfaces (Lawhern et al., 2018; Craik et al., 2019; Kundu and Ari, 2020; Zhang et al., 2021) decoding finger kinematics (Xie et al., 2018; Petrosyan et al., 2021) and seizure detection (Gao et al., 2020). Recently, it was shown that CNN outperformed linear decoder in estimating EMG signals from neural signals recorded from the spinal cord (Guo et al., 2018). In the current study, we used a 3D-CNN with a regression layer to decode the hindlimb joint kinematics from neural signals recorded from the lateral column as well as from the dorsal column. We also compared the performance of the 3D-CNN with conventional decoding models such as partial least squares (PLS) (Geladi and Kowalski, 1986) and Lasso (Zou and Hastie, 2005).

## Materials and Methods

The animals used in this study were kept and handled in compliance with the Experimental Animal Regulation Ordinances defined by the Ministry of Health and Medical Education, IRAN. All surgical procedures and experimental protocols involving animal models described in this paper were approved by the Institutional Animal Care and Ethics Committee of the Iran Neural Technology Research Center, Iran University of Science and Technology. All protocols and methods were performed according to the recommendations and relevant guidelines for the care and use of laboratory animals.

### Microwire Array Fabrication

**FIGURE 1:**
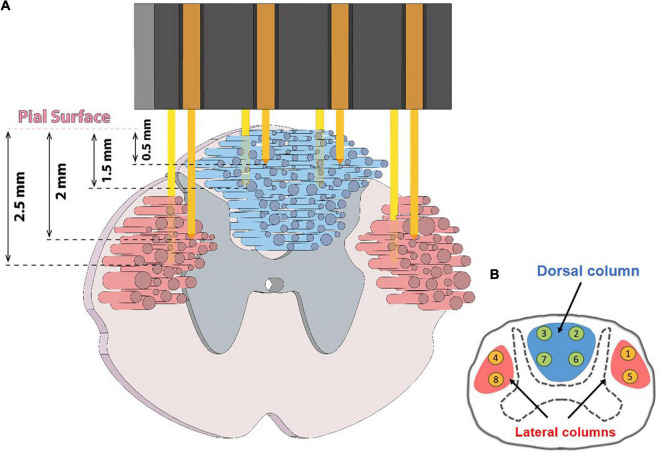
**(A)** Schematic figure showing ascending and descending tracts in the spinal cord and how and where the microwire array was implanted. **(B)** Numbering of eight implanted electrodes in the dorsal and lateral columns.

Neural signals were recorded from the spinal cord using a custom-made microelectrode array. The array was fabricated using PFA-insulated tungsten wires with a diameter of 50 μm (No. 795500, A-M Systems, United States). As depicted in Figure 1, eight microwires were arranged in two rows with an inter-electrode spacing of ∼1.5 mm in each row to cover the distance from left to right lateral columns. In each row, the length of the microwires that should be inserted in the lateral columns of the spinal cord were considered longer than those that should be inserted in the dorsal column of the spinal cord (∼ 0.5–1.5 mm length for the dorsal column electrodes and 1.5–2.5 mm for the lateral column). The lengths of the microwires of the second row were considered to be about ∼ 0.5 mm longer than the first row to cover different depths.

### Implantation Process

The experiments were conducted on two male cats (3.1–4.2 kg). Prior to surgery, the cats were trained for approximately 2 months to walk on the treadmill. We encouraged the animals to walk by rewarding with the pellet food. Initial anesthesia was induced by intramuscular injection of ketamine (20 mg/kg) into the cranial thigh muscle. The animals were then intubated with endotracheal tube and maintained at a surgical level of anesthesia under isoflurane inhalation (1.0–3.0% in O2). A partial 4 × 4 mm laminectomy was performed over the left (with respect to the rostral-caudal vector) L4 vertebra. Four screws were inserted into the bone of the L4 vertebra in the bulkier regions. We utilized these screws as anchors for the array and connector. The array and connector were fixed to the vertebra using dental acrylic.

The animal was then placed on a stereotactic frame (SN-1N, Narishige Group Product, Japan). The electrode array connector was attached to a micromanipulator (SM-15, Narishige Group Product, Japan) that could control the three-dimensional positioning of the electrode with a minimum graduation of 10 μm. To implant the electrode, a 4 × 4 mm section of the dura mater below the L4 vertebra was gently opened with an iridectomy scissor to obtain direct access to the spinal cord. Then, the array was vertically inserted into the target position in the spinal cord to cover the dorsal column and both the left and right lateral columns (Figure 1). After implantation, the surface of the spinal cord was covered with a thin layer of Sylgard 184. Then, the array and connector were attached to the bone using metal screws and dental acrylic. The DC and LC, at the L3–L4 spinal segments, typically cover a span of 0.0–1.5 and 1.5–3.0 mm laterally from the midline to the lateral side and a span of 0.0–1.5 and 1.5–3.0 mm in depth from the dorsal surface, respectively. The microelectrode array was implanted in the range 0.5–2.5 mm laterally from the midline and 0.5–2.5 mm in depth from the dorsal surface to cover the DC and LC. A tungsten reference wire was placed proximally in the epidural space of the spinal canal.

After surgery, necessary care, such as regular injection of Cefazolin and tramadol were taken to reduce the risk of infection and to relieve pain. Cefazolin (30 mg/kg) was intramuscularly injected twice daily for a week and tramadol (2 mg/kg) daily for 2 or 3 days after surgery. The urination and excretion of the animals were monitored daily to check for possible abnormalities.

### Data Collection

**FIGURE 2:**
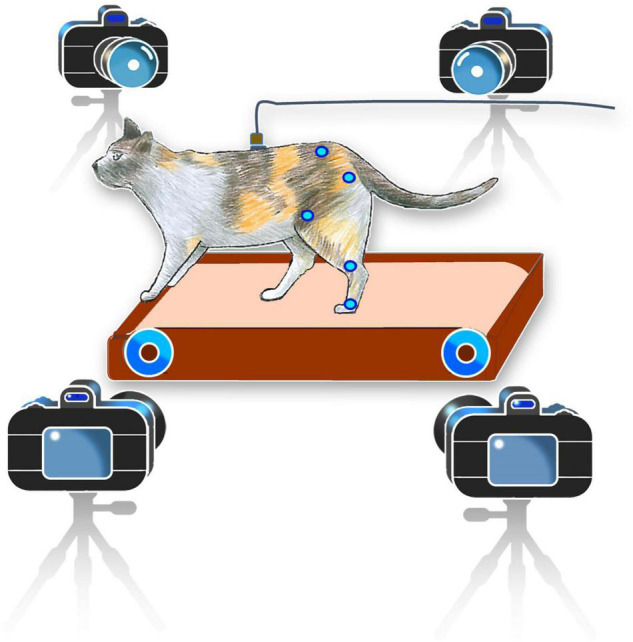
Schematic representation of the experimental setup. Cats were trained to walk on the treadmill in a hindlimb-only condition while keeping their forelimbs on the front frame of the treadmill for balance. Kinematics information was recorded using a motion capture system. Neural signals were recorded during walking on a treadmill and converted from analog to digital using a data acquisition system and were then used to decode the joint angles in a desktop computer.

The neural signal and kinematics of the hindlimbs were collected almost once a week. On each experimental day, one to three recording sessions were conducted. In cat 1, 18 sessions of the experiment were conducted for 14 weeks and in cat 2, 10 sessions were conducted. The duration of each recording session was 2–3 min. On average, each cat took 40 steps per minute. During each session, the animals could walk on a running treadmill with their hindlimbs only while keeping their forelimbs on the front frame of the treadmill (Figure 2). It should be noted that locomotor pattern resulted during hindlimb-only locomotion with placing forelimbs on a stationary platform is different from that resulted during normal quadrupedal locomotion (Harnie et al., 2022). The walking cadence of cat 1 varied between 0.59 and 0.8 steps per second, while the walking cadence of cat 2 had less variation between 0.9 and 0.99 steps per second.

### Joint Angle Measurements

**FIGURE 3:**
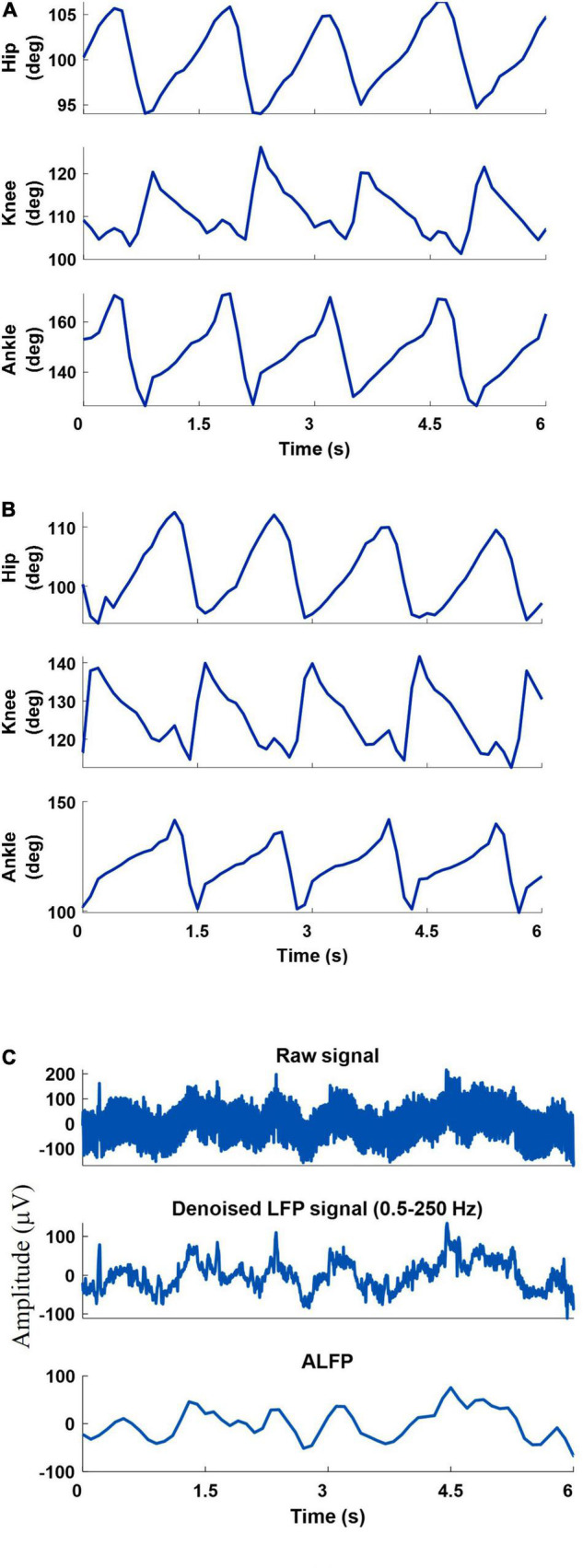
An example of the recorded joint angles from the left leg **(A)** and right leg **(B)**, recorded raw signal, filtered raw signal (0.5-250 Hz), and amplitude of the filtered LFP signal **(C)**.

A 6-camera motion capture system (Vicon Motion Systems Ltd., United Kingdom) was used to measure the joint angles at a sampling rate of 50 Hz. To measure the hindlimb joint angles of both legs, ten reflective markers were attached to the iliac crest, greater trochanter (hip joint), lateral condyle of the femur (knee joint), lateral malleolus (ankle joint), and the distal end of the fifth metatarsophalangeal) joints of both legs. Three-dimensional positions of markers were obtained using a motion capture software (Tracker 2.2, Vicon, United Kingdom). Using the measured positions of the five markers of each leg, three joint angles (ankle, knee, and hip) were calculated and displayed online using a custom-built LabVIEW program. Figure 3 shows an example of the recorded joint angles from the left and right leg.

### Neural Signals Recording and Preprocessing

**FIGURE 4:**
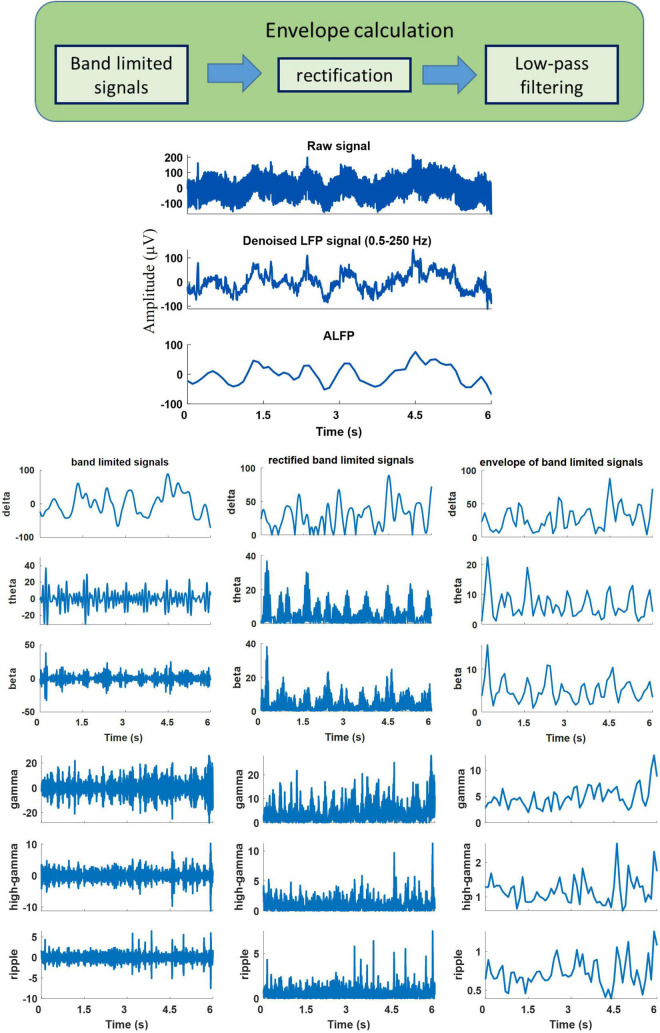
An example of the recorded raw signal, corresponding extracted local field potential (LFP), extracted band-limited signals at different frequency bands, corresponding rectified signals, and their envelopes.

**FIGURE 5:**
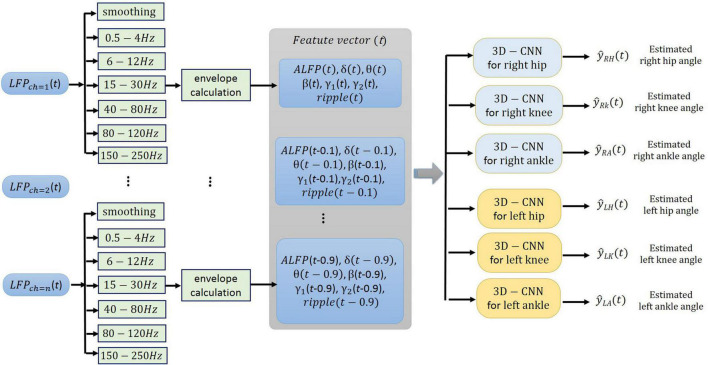
Schematic illustration of the neural signal processing. The smoothed amplitude of LFP signal (ALFP), envelopes of LFP signals at six frequency bands including δ (0.5-4 Hz), θ (6-12 Hz), β (15-30 Hz), γ (40-80 Hz), high-γ (80-120 Hz), and ripple (150-210 Hz), and their ten time lags (*t*,*t*−100*ms*,…,*t*−900*ms*) constitute the three-dimensional feature matrix [channel × time lag × (ALFP + average of signal envelope in each frequency band)].

The neural signals were measured using a multi-channel recording system (USB-ME64 system, Multichannel Systems Reutlingen, Germany) at a sampling rate of 500 Hz. Both the joint angles and the neural signals were synchronously captured using a custom-built LabVIEW (National Instruments, Austin, TX) program and saved for offline processing. Power line interference was removed using 4th order Butterworth notch filters at 50, 100, and 150 Hz. The signals were then band-pass filtered into six frequency bands using 4th order Butterworth filters. The frequency bands consisted of δ (0.5–4 Hz), θ (6–12 Hz), β (15–30 Hz), γ (40–80 Hz), high-γ (80–120 Hz), and ripple (150–210 Hz). Then, the envelope of the signal at each frequency band was computed by low-pass filtering (4th order Butterworth with a cutoff frequency of 4 Hz) the rectified band-limited signal and then resampled at 10 Hz (Figure 4). In addition, the average amplitude of the local field potential (ALFP) signal was also calculated by taking the average of the LFP signal within sliding windows of 200-ms length and 100-ms step. Finally, ten time lags of the envelope of the signal at each frequency band and ten time lags of the average amplitude of the LFP signal at each time *t* (*t*,*t*−100*ms*,*t*−900*ms*) were used as the features for decoding the kinematics information (Figure 5). The feature space can be constituted by a three-dimensional matrix (channel×lag×signal envelope at six frequency bands and ALFP features). This 3D feature matrix at each time was fed to the 3D-CNN to estimate the hindlimb joint angle.

### Decoding Model

**FIGURE 6:**
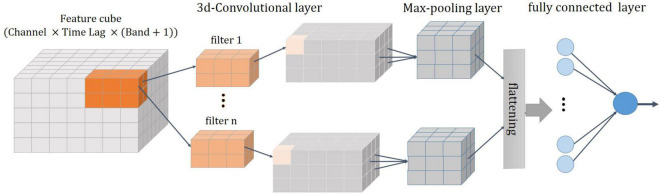
A schematic of 3D-CNN. It consists of four layers: a 3D convolutional layer, followed by a max-pooling layer, a flattering layer, and a fully connected layer.

The architecture of the 3D-CNN proposed in this work for decoding is shown in Figure 6. It consists of four layers: a 3D convolutional layer, followed by a pooling layer, a fully connected layer, and a regression layer (Patterson and Gibson, 2017). The features described in the previous section were organized in a three-dimensional array [channel×time lag×(ALFP and average of signal envelope in each frequency band)] and then fed into the 3D convolutional layer. The convolution layer is the fundamental component of the CNN that uses the local correlation of the information in the input to extract features. The process of the convolution operation is convolving a filter (or a kernel) to the input and the result is a feature map (activation map). The filter is applied across the input volume in a sliding window manner. The filter out is computed at each position of the input by the dot product of the filter and the input window. This procedure is repeated with many different filters to form different feature maps. Then, a nonlinear activation function is applied to the convolution-layer output. The rectified linear unit (ReLU) is considered as the activation function of the neurons in this layer. Two key parameters of the convolution operation are size and number of filters. In this study, a total of 70 filters of size 7 × 6 × 8 are convolved into the input feature space with stride sizes of 1 in each dimension. The size of the filter and the number of filter are heuristically chosen to achieve the best classification performance. The distance that the window moves is called a stride. The common choice of a stride is 1. The convolutional layer followed by a pooling layer which reduces the dimensionality of the feature maps. The most common pooling operation is the max-pooling which extracts patches from the feature map and select the maximum value in each patch. Here, a max-pooling with a filter size of 2 × 2 × 2 and a stride of 1 was used. The output of the pooling layer is flattened (i.e., transformed into a one-dimensional array) and connected to a fully connected layer. Here, fully connected layer is a single hidden neural network with one output neuron. The output is the estimated joint angle.

Training a network is a process of estimating the weights of filters in convolution layer and weights in fully connected layers. The training is performed by minimizing a loss function which is of the mean squared error of estimation (differences between output predictions and measured values). The stochastic gradient descent with momentum solver is considered for minimizing the loss function and estimating the network. A regularization term for weights with a coefficient of 0.3 is also added to the loss function. The momentum that specifies the contribution of the last estimated parameter for the current parameter update is set to 0.75. Other training parameters, such as the mini-batch size and maximum number of epochs, are set to 128 and 10, respectively (Beale et al., 2017). These parameters are set based on the trial-and-error method and remained unchanged during the evaluation of all data.

### Performance Evaluation

To assess the performance of the CNN in decoding the hindlimb kinematics, the coefficient of determination, *R*^2^ value, was used. The *R*^2^can be defined as:


(1) $$R^{2}\left(\%\right)=\left(1-\frac{\sum_{\text{n}=1}^{\text{N}}{\left(y_{n}-{\hat{y}}_{n}\right)}^{2}}{\sum_{\text{n}=1}^{\text{N}}{\left(y_{n}-\bar{y}\right)}^{2}}\right)\times 100$$

where *y* is the measured joint angle, $\bar{y}$ is its mean value, $\hat{y}$ is the estimated angle, and *N* is the number of data points. Threefold cross-validation was used to train and test the model using the data recorded during each session of the experiment. In threefold cross validation, the dataset was split into three equal parts (they are called folds). Two fold of the data (67%) obtained during each session of the experiment was assigned for training and the remaining fold (33% of the data) were assigned for testing the model. In the next round of threefold cross-validation, a different fold of data was selected for testing and the remaining folds for training. The average of decoding performance over the threefold and all the sessions of the experiment were reported. Moreover, mutual information (MI) was employed to evaluate the information content of the recorded neural signals. MI measures the mutual dependence of two or more variables. In other words, MI is a measure of the information that a random signal have on the other random signals and can be quantified using entropy. If two random variables are independent, then their mutual information is zero, but if the mutual information is large, it means two variables are closely related. Mutual information was calculated based on an adaptive partitioning of the observation space (Darbellay and Vajda, 1999). MI has an advantage over the linear correlation coefficient in that it is able to capture both linear and nonlinear dependencies. MI was calculated between the extracted features from the LFP features at each frequency band and hindlimb kinematics. Analysis of variance (ANOVA) followed by Tukey’s HSD post hoc test was used to assess the statistical significance of the results and differences. A confidence level of 95% (*p* < 0.05) was chosen to indicate a significant difference. All data analyses were performed with customized algorithms written in MATLAB.

## Results

### Time-Frequency Analysis

**FIGURE 7:**
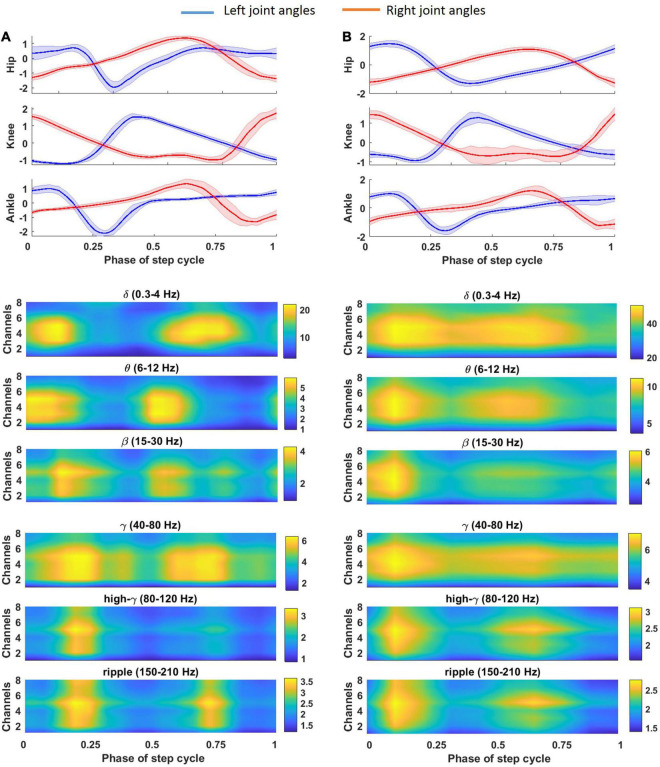
Average of gait cycle for the left **(A)** and right **(B)** leg during a typical trial of cat locomotion (cat 1, 56 days post-implant) on the treadmill and corresponding spatial time-frequency analysis of the recorded LFP signal. Average of the LFP envelope over different gait cycles is calculated for each channel and each frequency band.

The average of the gait cycle for the left and right legs during a typical trial of cat locomotion on the treadmill and the corresponding spatial time-frequency analysis of the recorded LFP signals are plotted in Figure 7A. It can be seen that a strong amplitude enhancement or event-related synchronization (ERS) and a strong amplitude attenuation or event-related desynchronization (ERD) occurred during joint flexion and extension, respectively. ERS and ERD occurred in both the dorsal column and lateral columns. ERS was observed in the ripple and high-γ bands during left hip flexion; β and γ bands during left ankle flexion; θ and β during left knee flexion; δ and β bands during right knee flexion; and δ and γ bands during right hip and ankle flexion. A short ERS in the ripple band at 70% of the gait cycle can be associated with a small right knee flexion. Figure 7B shows the average of the time-frequency over all sessions of the experiment. Almost, the same ERS/ERD patterns were observed in the averaged time-frequency across all sessions but with broader distributed ERS pattern.

### Mutual Information Analysis

**FIGURE 8:**
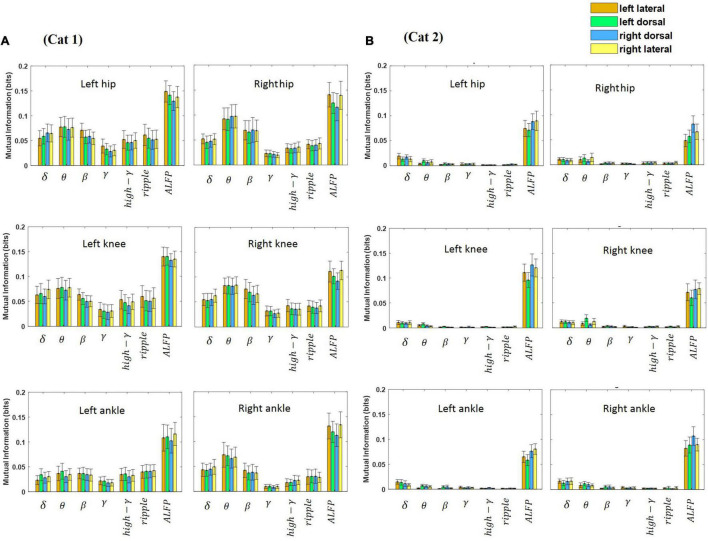
Average of mutual information between each feature and each joint angle (i.e., hip, knee, and ankle) for the left and right legs. The features were extracted from each channel and consisted of the LFP envelope at different frequency bands and the amplitude of LFP signal. The error bar on each bar graph represents the standard deviation. The results were obtained from cat 1 **(A)** and cat 2 **(B)**.

The relative kinematic information of the LFP signal recorded from the lateral and dorsal columns during treadmill locomotion was investigated. Hence, MI was calculated between each joint angle and each band-limited LFP signal recorded from each electrode for all sessions of experiment (18 sessions for cat 1 and 10 sessions for cat 2) and then averaged over all sessions. All the data obtained during each session of experiment (from beginning of treadmill locomotion to termination) were used for calculating MI. Moreover, the MI between the ALFP and each joint angle was computed. Figure 8 shows the average of the MI over the different sessions of the experiment and over the three joint angles at each frequency band for both legs and two cats. The results of the two-way ANOVA show that the ALFP contains significantly more information about limb kinematics than the frequency bands (*p* < 10e-7) in both cats. Comparing the information content of the frequency bands, the results show that the θ band provides significantly more information than the other frequency bands (*p* < 10e-7) in cat 1, and the δ and θ bands were more informative than the other bands (*p* < 0.01) in cat 2. Moreover, it was found that the kinematics information of different spinal regions (i.e., left and right lateral columns, left and right dorsal columns) are not significantly different. Comparison between the information of channels in the left and right sides of the spinal cord showed that there was no significant difference between the two sides of the spinal cord, except for cat 2, where the ALFP at the right side of the spinal cord provides significantly greater MI for both legs (*p* < 0.01).

**FIGURE 9:**
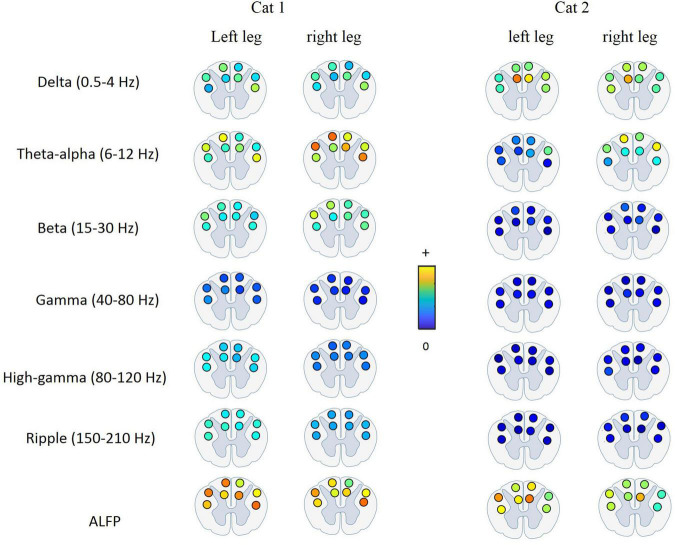
Average of mutual information between the LFP signal at each frequency band and each joint angle for different electrode positions in the spinal cord. The most kinematic information is represented by the low-frequency components. There is no significant difference between the kinematic information of the signals recorded from the DC and LC regions.

Figure 9 shows the average of MI between the LFP and hindlimb kinematics at different frequency bands for each recording channel and for both legs and both cats. It can be seen that the most kinematic information is represented by the low-frequency components. The results of the statistical test show that there is no significant difference between the kinematic information of the recording channels. This result is consistent with the previous study (Fathi and Erfanian, 2021) that the results show that there is no significant difference between the information content of the signals recorded from the DC and LC regions during walking on the treadmill.

### Effects of Walking Cadence

The effects of running speed on the oscillatory behavior of LFP signals recorded from the brain during locomotion have been investigated (Ahmed and Mehta, 2012; Bender et al., 2015; Noga et al., 2017). Increased running speed has been reported to be accompanied by hippocampal gamma oscillations (Ahmed and Mehta, 2012) and hippocampal theta oscillations (Bender et al., 2015). It was also demonstrated that theta LFP oscillations in the mesencephalic locomotor region are correlated with the speed of locomotion (Noga et al., 2017).

**FIGURE 10:**
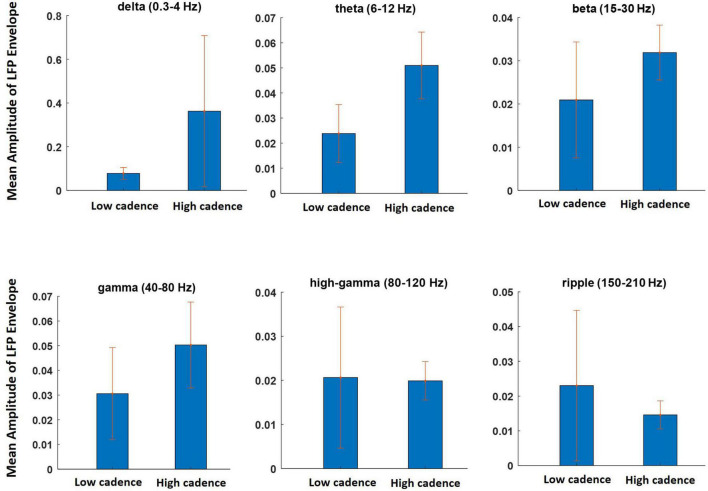
The effect of walking cadence on the mean amplitude of LFP envelope at various frequency bands during movement on the treadmill. The sessions on which animal walking cadence was between 0.59 and 0.67 steps per second were considered as lower cadence category while sessions with cadence between 0.73 and 0.8 steps per second considered as higher cadence category.

In this section, the effects of the walking cadence on the LFP and the spectral features are investigated. The data from different sessions were divided into two subgroups: one with walking cadence lower than a lower threshold (0.67 steps per second) and the other with higher than the threshold (0.73 steps per second). The data obtained during 12 sessions of experiment (6 sessions for low cadence and 6 sessions for high cadence) were selected for this analysis. We refer to these two groups as the lower and higher cadence groups, respectively. The mean envelope values of the LFP signals at different frequency bands were computed for both groups. Figure 10 shows the results of the analysis. It can clearly be seen that the amplitude of the low-frequency components (i.e., delta, theta, beta, and gamma) increased during the high cadence of locomotion relative to that of the low cadence. However, this increase was not observed for the high-gamma and ripple components. The results of this analysis indicate that the LFP oscillations can reflect running speed. The results show that there is a significant difference between the mean amplitude of LFP envelope obtained during low cadence and high cadence walking only at the theta frequency band (*p* = 0.03).

### Decoding and Its Performance

**FIGURE 11:**
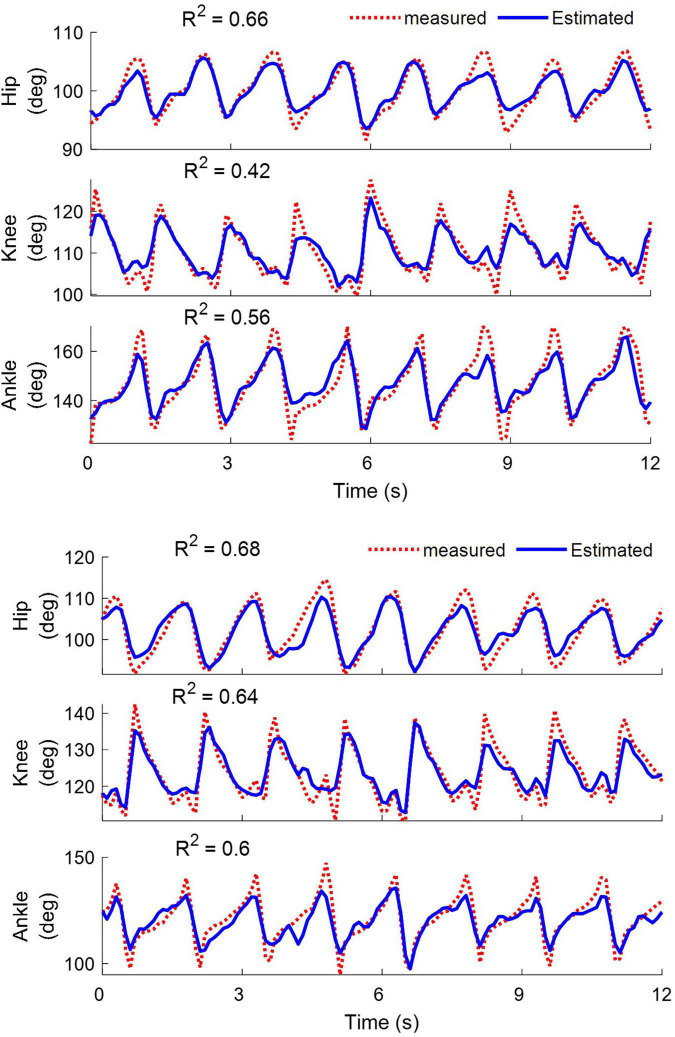
Examples of decoding the hip, knee, and ankle joint angles for the left **(top)** and right leg **(bottom)** during walking on the treadmill. The results are obtained from cat 1 on day 12 after implantation. The coefficient of determination (*R*^2^) values obtained for the left leg are 0.66, 0.42, and 0.56 for the hip, knee and ankle angles, respectively. For the right leg, the *R*^2^ values are 0.68, 0.64, and 0.6 for the hip, knee, and ankle angles, respectively.

Figure 11 shows a typical example of decoding the joint angles in cat 1 while walking on the treadmill for the left (top) and right (bottom) legs using the neural signals recorded from all electrodes implanted in the spinal cord. It can be seen that good tracking performance was obtained for all joints of two legs during hindlimb-only locomotion. The coefficients of determination (*R*2) obtained for the hip, knee, and ankle joints of the left leg were 0.66, 0.42, and 0.56, respectively, and those for the right leg were 0.68, 0.64, and 0.60, respectively.

**FIGURE 12:**
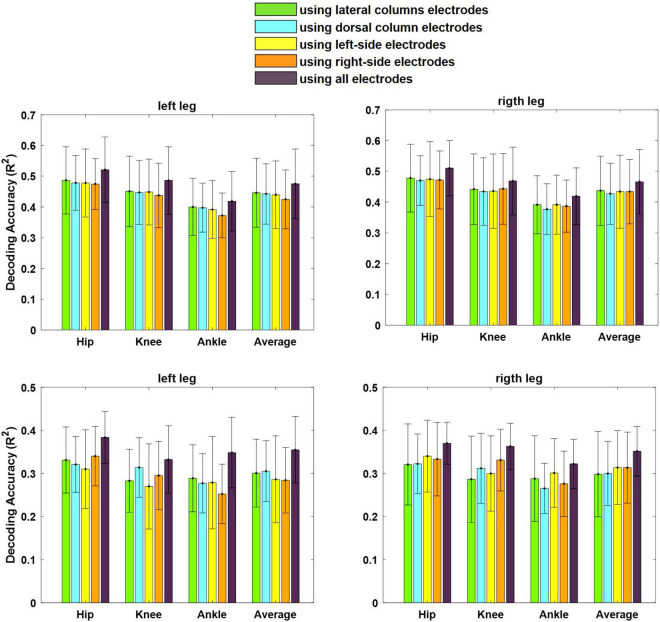
Average of decoding performance for both left and right legs using the LFP signals recorded from electrodes implanted in the lateral columns (electrodes 1, 4, 5, and 8), dorsal columns (electrodes 2, 3, 6, and 7), left-side of the spinal cord (electrodes 3, 4, 7, and 8), right-side of the spinal cord (electrodes 1, 2, 5, and 6), and all eight electrodes. The error bar on each bar graph represents the standard deviation. The results are obtained from cat 1 (top) and cat 2 (bottom).

Figure 12 shows the average of the decoding performance for the left and right legs using the neural signals recorded from the left and right lateral columns (channels 1, 4, 5, and 8) as well as from the left and the right dorsal columns (channels 2, 3, 6, and 7) and from all channels. The results of the statistical analysis show that there is no significant difference (*p* > 0.4) between the performances obtained using the neural signals recorded from the lateral columns and that from the dorsal column for both cats. This result indicates that the lateral column can be used as the source of extracting motor information for motor neuroprosthesis.

Furthermore, there was no significant difference between the decoding performance of the left and right legs using the neural signals recorded from the right side of the spinal cord (*p* > 0.16) (channels 1, 2, 5, and 6) as well as from the left side (*p* > 0.27) (channels 3, 4, 7, 8). Moreover, using all the channels in decoding could improve the decoding performance with *p* < 0.05 for cat 1 and with *p* < 0.001 for cat2.

### Decoding Model Comparison

**FIGURE 13:**
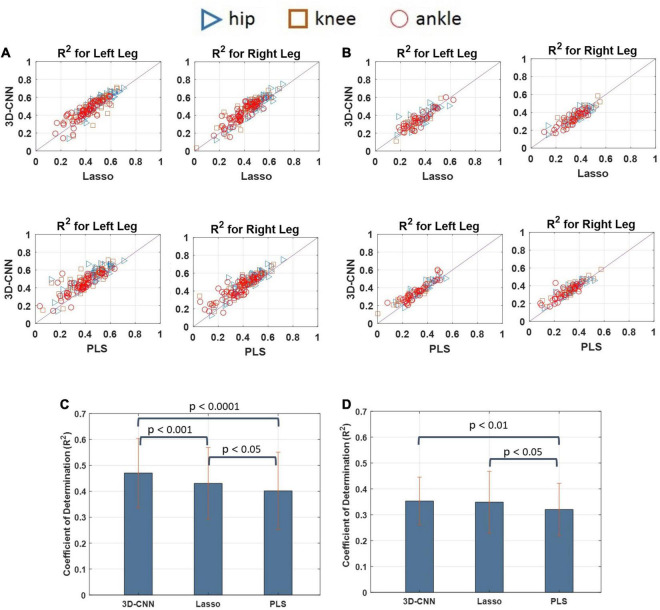
Scatter plots comparing the decoding performances obtained by the 3D-CNN, Lasso, and PLS for cat 1 **(A)** and cat 2 **(B)**. Each point in the scatter plot represents the decoding performance obtained in one trial of experiment. The results over the diagonal line indicate that the method in y-axis (i.e., 3D-CNN) outperforms the method in x-axis (e.g., PLS or Lasso). Pairwise comparisons between the decoding performance obtained using different methods for cat 1 **(C)** and cat 2 **(D)**. The results show that the 3D-CNN yields higher decoding performance than the Lasso and PLS methods.

The decoding performance of the 3D-CNN was also compared with that obtained using a conventional method such as the Lasso and PLS regression models. The decoding performance of the 3D-CNN was also compared with that obtained using a conventional method such as the Lasso and PLS regression models. The CNN was implemented using MATLAB with Deep Learning Toolbox and PLS as well as Lasso with Statistics and Machine Learning Toolbox. Pairwise comparisons of the decoding performance using scatterplots are provided in Figures 13A,B for cats 1 and 2, respectively. For cat 1, the decoding performance obtained using the 3D-CNN model outperformed both the Lasso and PLS methods. For cat 2, it can be seen that the 3D-CNN outperformed the PLS, but no clear difference was observed when comparing the 3D-CNN with the Lasso method. Figures 13C,D show the average decoding performance over the left and right legs for each cat. One-way ANOVA with Tukey’s HSD post hoc test was performed to compare the decoding performance using different methods. The results of statistical tests showed that the 3D-CNN outperformed the Lasso (*p* < 0.001) and PLS (*p* < 0.0001) in cat 1. In cat 2, the *R*^2^-values obtained using the 3D-CNN were significantly higher than those obtained using the PLS (*p* < 0.01), while no such difference exists between the 3D-CNN and Lasso.

## Discussion and Conclusion

In this study, we explored the capability of descending and ascending spinal cord neural signals in decoding both hindlimb kinematics. The decoding of the hindlimb kinematics information from the neural signals recorded from the descending tracts within the spinal cord indicates that the motor information could be extracted from these neural signals to develop motor neuroprostheses.

Here, we demonstrated that the neural signals recorded from the lateral columns can decode hindlimb kinematics as accurately as the dorsal columns. Interestingly, the neural signals from one side of the spinal cord could be used to decode both ipsilateral and contralateral hindlimb movements with very similar accuracy.

Decoding the ipsilateral and contralateral hindlimb movements indicates that the lateral columns contain information about the motor behaviors of discrete body parts. It has been reported that excitatory spinal interneurons are responsible for the timing and amplitude control of locomotor movements (Bouvier et al., 2015; Hayashi et al., 2018). Here, we showed that the lateral columns contain information about the timing and amplitude of the ipsilateral and contralateral movements. This finding suggests that supraspinal centers contribute to the locomotor coordination of timing and amplitude (Armstrong, 1986; Drew and Marigold, 2015; Frigon, 2017). In the current study, the results of the time-frequency analysis showed that the frequency bands of the LFP signals recorded from the lateral columns could represent the onset and offset of locomotor activity.

Although, the efferent signal carries motor information which is different from sensory information, the analysis of MI shows that there is no significant difference between hindlimb kinematics information obtained from the lateral columns and from the dorsal column during ipsilateral and contralateral movements. Interestingly, this result is in agreement with the results of decoding performance. The results of decoding show that there is no significant difference (*p* > 0.4) between the performances obtained using the neural signals recorded from the lateral columns and that from the dorsal column for both cats. Moreover, the MI analysis shows that the theta band provides significantly more information about the hindlimb kinematics than the other frequency bands (*p* < 10e-7). Previous studies have demonstrated that theta frequency oscillations of the hippocampal LFP are the main brain rhythm influenced by the periodic movements of locomotion (Ledberg and Robbe, 2011; Noga et al., 2017). Interestingly, our results also showed that the theta rhythm of the spinal LFP was associated with locomotor activity. It has also been shown that theta and gamma oscillations in the hippocampal increase in amplitude and frequency with faster running speed (Ledberg and Robbe, 2011; Ahmed and Mehta, 2012; Bender et al., 2015). Our primary results show that the power in different frequency bands changes with locomotion speed. In particular, the theta power increased with a higher locomotion speed. However, further experiments are required to provide further evidence.

Furthermore, we employed a state-of-the-art decoding model, 3D-CNN, to decode the hindlimb joint angles from the descending and ascending neural signals. The results show significant improvements in comparison to other well-known regression models in the field, such as the PLS and Lasso methods. However, the performance could still be increased by searching for optimal parameters or using deeper structures that are beyond the scope of the current study and will be considered for future work.

In the current study, we used the LFPs of the ascending and descending tracts to decode the hindlimb kinematics during cat locomotion on the treadmill, whereas in the previous study (Fathi and Erfanian, 2019), spike activity recorded from the dorsal horn was used to decode the kinematics information during passive hindlimb movement. Here, we face an important unanswered question: which one of these spinal neural signals; LFPs or multi-unit spikes, is the most suitable for decoding hindlimb kinematics considering longevity of recording, movement artifact, and computational cost. Comparisons between the intracortical LFPs and multi-unit spikes have been performed by a number of groups (Jackson and Hall, 2017) and showed that LFPs were significantly more stable than spikes (Flint et al., 2016), however, since the spinal cord environment is tougher than the brain for recording, the intraspinal recording faces more severe challenges than intracortical recording and can be considered for future research purposes.

Furthermore, the results presented in the current study are based on the data obtained from two cats. To make broad conclusions, the experiments should be conducted on more cats which can be considered for future study.

## Data Availability Statement

The raw data supporting the conclusions of this article will be made available by the authors, without undue reservation.

## Ethics Statement

The animals used in this study were kept and handled in compliance with the Experimental Animal Regulation Ordinances defined by the Ministry of Health and Medical Education, Iran. All surgical procedures and experimental protocols involving animal models described in this manuscript were approved by the Institutional Animal Care and Ethics Committee of the Iran Neural Technology Research Center, Iran University of Science and Technology. All protocols and methods were performed according to the recommendations and relevant guidelines for the care and use of laboratory animals.

## Author Contributions

AE and YF contributed to the design of experiments. YF conducted the experiments and analyzed the data. Both authors contributed to manuscript preparation and revision.

## Conflict of Interest

The authors declare that the research was conducted in the absence of any commercial or financial relationships that could be construed as a potential conflict of interest.

## Publisher’s Note

All claims expressed in this article are solely those of the authors and do not necessarily represent those of their affiliated organizations, or those of the publisher, the editors and the reviewers. Any product that may be evaluated in this article, or claim that may be made by its manufacturer, is not guaranteed or endorsed by the publisher.

## References

[B1] Armstrong D. M. (1986). Supraspinal contributions to the initiation and control of locomotion in the cat. *Prog. Neurobiol.* 26 273–361. 10.1016/0301-0082(86)90021-3 3526411

[B2] Ahmed O. J. Mehta M. R. (2012). Running speed alters the frequency of hippocampal gamma oscillations. *J. Neurosci.* 32 7373–7383. 10.1523/JNEUROSCI.5110-11.2012 22623683 PMC3366345

[B3] Barroso F. O. Yoder B. Tentler D. Wallner J. J. Kinkhabwala A. A. Jantz M. K. (2019). Decoding neural activity to predict rat locomotion using intracortical and epidural arrays. *J. Neural Eng.* 16:36005. 10.1088/1741-2552/ab0698 30754031

[B4] Beale M. H. Hagan M. T. Demuth H. B. (2017). *MATLAB Neural Network Toolbox User’s Guide (version R2017b).* Natick, MA: MathWorks.

[B5] Bender F. Gorbati M. Cadavieco M. C. Denisova N. Gao X. Holman C. (2015). Theta oscillations regulate the speed of locomotion via a hippocampus to lateral septum pathway. *Nat. Commun.* 6:8521. 10.1038/ncomms9521 26455912 PMC4633825

[B6] Bonizzato M. Pidpruzhnykova G. DiGiovanna J. Shkorbatova P. Pavlova N. Micera S. (2018). Brain-controlled modulation of spinal circuits improves recovery from spinal cord injury. *Nat. Commun.* 9:3015. 10.1038/s41467-018-05282-6 30068906 PMC6070513

[B7] Bouvier J. Caggiano V. Leiras R. Caldeira V. Bellardita C. Balueva K. (2015). Descending command neurons in the brainstem that halt locomotion. *Cell* 163 1191–1203. 10.1016/j.cell.2015.10.074 26590422 PMC4899047

[B8] Bruns T. M. Wagenaar J. B. Bauman M. J. Gaunt R. A. Weber D. J. (2013). Real-time control of hind limb functional electrical stimulation using feedback from dorsal root ganglia recordings. *J. Neural Eng.* 10:026020. 10.1088/1741-2560/10/2/026020 PMC3640462 23503062

[B9] Capogrosso M. Milekovic T. Borton D. Wagner F. Moraud E. M. Mignardot J.-B. (2016). A brain–spine interface alleviating gait deficits after spinal cord injury in primates. *Nature* 539 284–288. 10.1038/nature20118 27830790 PMC5108412

[B10] Craik A. He Y. Contreras-Vidal J. L. (2019). Deep learning for electroencephalogram (EEG) classification tasks: a review. *J. Neural Eng.* 16:31001. 10.1088/1741-2552/ab0ab5 30808014

[B11] Darbellay G. A. Vajda I. (1999). Estimation of the information by an adaptive partitioning of the observation space. *IEEE Trans. Inf. Theory* 45 1315–1321. 10.1109/18.761290

[B12] Debnath S. Bauman M. J. Fisher L. E. Weber D. J. Gaunt R. A. (2014). Microelectrode array recordings from the ventral roots in chronically implanted cats. *Front. Neurol.* 5:104. 10.3389/fneur.2014.00104 25071697 PMC4083189

[B13] Drew T. Marigold D. S. (2015). Taking the next step: cortical contributions to the control of locomotion. *Curr. Opin. Neurobiol.* 33 25–33. 10.1016/j.conb.2015.01.011 25643847

[B14] Fathi Y. Erfanian A. (2019). A probabilistic recurrent neural network for decoding hind limb kinematics from multi-segment recordings of the dorsal horn neurons. *J. Neural Eng.* 16:036023. 10.1088/1741-2552/ab0e51 30849772

[B15] Fathi Y. Erfanian A. (2021). Decoding hindlimb kinematics from descending and ascending neural signals during cat locomotion. *J. Neural Eng.* 18:26015. 10.1088/1741-2552/abd82a 33395669

[B16] Flint R. D. Scheid M. R. Wright Z. A. Solla S. A. Slutzky M. W. (2016). Long-term stability of motor cortical activity: implications for brain machine interfaces and optimal feedback control. *J. Neurosci.* 36 3623–3632. 10.1523/JNEUROSCI.2339-15.2016 27013690 PMC4804017

[B17] Fitzsimmons N. Lebedev M. Peikon I. Nicolelis M. (2009). Extracting kinematic parameters for monkey bipedal walking from cortical neuronal ensemble activity. *Front. Integr. Neurosci.* 3:3. 10.3389/neuro.07.003.2009 19404411 PMC2659168

[B18] Frigon A. (2017). The neural control of interlimb coordination during mammalian locomotion. *J. Neurophysiol.* 117 2224–2241. 10.1152/jn.00978.2016 28298308 PMC5454475

[B19] Frigon A. Rossignol S. Frigon A. (2011). Recovery of locomotion after spinal cord injury?: some facts and mechanisms. *Annu. Rev. Neurosci.* 34 413–440. 10.1146/annurev-neuro-061010-113746 21469957

[B20] Gao Y. Gao B. Chen Q. Liu J. Zhang Y. (2020). Deep convolutional neural network-based epileptic electroencephalogram (EEG) signal classification. *Front. Neurol.* 11:375. 10.3389/fneur.2020.00375 32528398 PMC7257380

[B21] Geladi P. Kowalski B. (1986). Partial least-squares regression: a tutorial. *Anal. Chim. Acta* 185 1–17. 10.1016/0003-2670(86)80028-9

[B22] Gok S. Sahin M. (2019). Prediction of forelimb EMGs and movement phases from corticospinal signals in the rat during the reach-to-pull task. *Int. J. Neural Syst.* 29:1950009. 10.1142/S0129065719500096 31111753

[B23] Guo Y. Foulds R. A. Adamovich S. V. Sahin M. (2014). Encoding of forelimb forces by corticospinal tract activity in the rat. *Front. Neurosci* 8:62. 10.3389/fnins.2014.00062 24847198 PMC4013477

[B24] Guo Y. Gok S. Sahin M. (2018). Convolutional networks outperform linear decoders in predicting EMG from spinal cord signals. *Front. Neurosci.* 12:689. 10.3389/fnins.2018.00689 30386200 PMC6199918

[B25] Han S. Chu J. U. Kim H. Park J. W. Youn I. (2017). Multiunit activity-based real-time limb-state estimation from dorsal root ganglion recordings. *Sci. Rep.* 7 1–14. 10.1038/srep44197 28276474 PMC5343572

[B26] Hansen M. Haugland M. K. Sinkjær T. (2004). Evaluating robustness of gait event detection based on machine learning and natural sensors. *IEEE Trans. Neural Syst. Rehabil. Eng.* 12 81–88. 10.1109/TNSRE.2003.819890 15068191

[B27] Harnie J. Audet J. Mari S. Lecomte C. G. Merlet A. N. Genois G. (2022). State-and condition-dependent modulation of the hindlimb locomotor pattern in intact and spinal cats across speeds. *Front. Syst. Neurosci* 16:814028. 10.3389/fnsys.2022.814028 35221937 PMC8863752

[B28] Hayashi M. Hinckley C. A. Driscoll S. P. Moore N. J. Levine A. J. Hilde K. L. (2018). Graded arrays of spinal and supraspinal V2a interneuron subtypes underlie forelimb and hindlimb motor control. *Neuron* 97 869.e–884.e. 10.1016/j.neuron.2018.01.023 29398364 PMC8601153

[B29] Holinski B. J. Everaert D. G. Mushahwar V. K. Stein R. B. (2013). Real-time control of walking using recordings from dorsal root ganglia. *J. Neural Eng.* 10:056008. 10.1088/1741-2560/10/5/056008 PMC3791106 23928579

[B30] Jackson A. Zimmermann J. B. (2012). Neural interfaces for the brain and spinal cord—restoring motor function. *Nat. Rev. Neurol.* 8 690–699. 10.1038/nrneurol.2012.219 23147846

[B31] Jackson A. Hall T. M. (2017). Decoding local field potentials for neural interfaces. *IEEE Trans. Neural Syst. Rehabil. Eng.* 25 1705–1714. 10.1109/TNSRE.2016.2612001 28113942 PMC6051483

[B32] Kashkoush A. I. Gaunt R. A. Weber D. J. (2019). Recording single- and multi-unit neuronal action potentials from the surface of the dorsal root ganglion. *Sci. Rep.* 9 1–12. 10.1038/s41598-019-38924-w 30808921 PMC6391375

[B33] Knudsen E. B. Moxon K. A. (2017). Restoration of hindlimb movements after complete spinal cord injury using brain-controlled functional electrical stimulation. *Front. Neurosci.* 11:715. 10.3389/fnins.2017.00715 29311792 PMC5742140

[B34] Kundu S. Ari S. (2020). MsCNN: a deep learning framework for P300-based brain–computer interface speller. *IEEE Trans. Med. Robot. Bionics* 2 86–93. 10.1109/TMRB.2019.2959559

[B35] Lawhern V. J. Solon A. J. Waytowich N. R. Gordon S. M. Hung C. P. Lance B. J. (2018). EEGNet: a compact convolutional neural network for EEG-based braincomputer interfaces. *J. Neural Eng.* 15:56013. 10.1088/1741-2552/aace8c 29932424

[B36] LeCun Y. Bengio Y. Hinton G. (2015). Deep learning. *Nature* 521 436–444. 10.1038/nature14539 26017442

[B37] Ledberg A. Robbe D. (2011). Locomotion-related oscillatory body movements at 6–12 Hz modulate the hippocampal theta rhythm. *PLoS One* 6:e27575. 10.1371/journal.pone.0027575 22102910 PMC3216955

[B38] Lemon R. N. (2008). Descending pathways in motor control. *Annu. Rev. Neurosci.* 31 195–218. 10.1146/annurev.neuro.31.060407.125547 18558853

[B39] Li W. Zhu L. Shi Y. Guo K. Cambria E. (2020). User reviews: sentiment analysis using lexicon integrated two-channel CNN–LSTM family models. *Appl. Soft Comput.* 94:106435. 10.1016/j.asoc.2020.106435

[B40] Li Y. Zhao J. Lv Z. Pan Z. (2021). Multimodal medical supervised image fusion method by CNN. *Front. Neurosci.* 15:303. 10.3389/fnins.2021.638976 34149344 PMC8206541

[B41] Liu D. Li Y. Lin J. Li H. Wu F. (2020). Deep learning-based video coding: a review and a case study. *ACM Comput. Surv.* 53:11. 10.1145/3368405

[B42] Micera S. Jensen W. Sepulveda F. Riso R. R. Sinkjær T. (2001). Neuro-fuzzy extraction of angular information from muscle afferents for ankle control during standing in paraplegic subjects: an animal model. *IEEE Trans. Biomed. Eng.* 48 787–794. 10.1109/10.930903 11442290

[B43] Mustaqeem Kwon S. (2020). A CNN-assisted enhanced audio signal processing for speech emotion recognition. *Sensors* 20:183. 10.3390/s20010183 31905692 PMC6982825

[B44] Noga B. R. Sanchez F. J. Villamil L. M. O’Toole C. Kasicki S. Olszewski M. (2017). LFP oscillations in the mesencephalic locomotor region during voluntary locomotion. *Front. Neural Circuits* 11:34. 10.3389/fncir.2017.00034 28579945 PMC5437718

[B45] Patterson J. Gibson A. (2017). *Deep Learning: A Practitioner’s Approach.* Sebastopol, CA: O’Reilly Media, Inc.

[B46] Petrosyan A. Sinkin M. Lebedev M. Ossadtchi A. (2021). Decoding and interpreting cortical signals with a compact convolutional neural network. *J. Neural Eng* 18:026019. 10.1088/1741-2552/abe20e 33524962

[B47] Prasad A. Sahin M. (2006). Extraction of motor activity from the cervical spinal cord of behaving rats. *J. Neural Eng.* 3 287–292. 10.1088/1741-2560/3/4/005 17124332 PMC2396534

[B48] Prasad A. Sahin M. (2010). Characterization of neural activity recorded from the descending tracts of the rat spinal cord. *Front. Neurosci.* 4:21. 10.3389/fnins.2010.00021 20589238 PMC2904587

[B49] Prasad A. Sahin M. (2012). Can motor volition be extracted from the spinal cord? *J. Neuroeng. Rehabil.* 9:41. 10.1186/1743-0003-9-41 22713735 PMC3443439

[B50] Rigosa J. Panarese A. Dominici N. Friedli L. van den Brand R. Carpaneto J. (2015). Decoding bipedal locomotion from the rat sensorimotor cortex. *J. Neural Eng.* 12:56014. 10.1088/1741-2560/12/5/056014 26331532

[B51] Rigosa J. Weber D. J. Prochazka A. Stein R. B. Micera S. (2011). Neuro-fuzzy decoding of sensory information from ensembles of simultaneously recorded dorsal root ganglion neurons for functional electrical stimulation applications. *J. Neural Eng.* 8:046019. 10.1088/1741-2560/8/4/046019 21701057

[B52] Riso R. R. Mosallaie F. K. Jensen W. Sinkjaer T. (2000). Nerve cuff recordings of muscle afferent activity from tibial and peroneal nerves in rabbit during passive ankle motion. *IEEE Trans. Rehabil. Eng.* 8 244–258. 10.1152/physiol.00042.2008 10896197

[B53] Rossignol S. Barrière G. Alluin O. Frigon A. (2009). Re-expression of locomotor function after partial spinal cord injury. *Physiology* 24 127–139. 10.1152/physiol.00042.2008 19364915

[B54] Song I. Chu J. U. Park S. E. Hwang D. Youn I. (2017). Ankle-angle estimation from blind source separated afferent activity in the sciatic nerve for closed-loop functional neuromuscular stimulation system. *IEEE Trans. Biomed. Eng.* 64 834–843. 10.1109/TBME.2016.2580705 27323354

[B55] Song W. Ramakrishnan A. Udoekwere U. I. Giszter S. F. (2009). Multiple types of movement-related information encoded in hindlimb/trunk cortex in rats and potentially available for brain–machine interface controls. *IEEE Trans. Biomed. Eng.* 56 2712–2716. 10.1109/TBME.2009.2026284 19605313 PMC2883457

[B56] Stein R. B. Weber D. J. Aoyagi Y. Prochazka A. Wagenaar J. B. M. Shoham S. (2004). Coding of position by simultaneously recorded sensory neurones in the cat dorsal root ganglion. *J. Physiol.* 560 883–896. 10.1113/jphysiol.2004.068668 15331686 PMC1665274

[B57] Takakusaki K. Chiba R. Nozu T. Okumura T. (2016). Brainstem control of locomotion and muscle tone with special reference to the role of the mesopontine tegmentum and medullary reticulospinal systems. *J. Neural Transm.* 123 695–729. 10.1007/s00702-015-1475-4 26497023 PMC4919383

[B58] Wagenaar J. B. Ventura V. Weber D. J. (2011). State-space decoding of primary afferent neuron firing rates. *J. Neural Eng.* 8:016002. 10.1088/1741-2560/8/1/016002 PMC3048170 21245525

[B59] Weber D. J. Stein R. B. Everaert D. G. Prochazka A. (2006). Decoding sensory feedback from firing rates of afferent ensembles recorded in cat dorsal root ganglia in normal locomotion. *IEEE Trans. Neural Syst. Rehabil. Eng.* 14 240–243. 10.1109/TNSRE.2006.875575 16792303

[B60] Weber D. J. Stein R. B. Everaert D. G. Prochazka A. (2007). Limb-state feedback from ensembles of simultaneously recorded dorsal root ganglion neurons. *J. Neural Eng.* 4 S168–S180. 10.1088/1741-2560/4/3/S04 17873416

[B61] Widiastuti N. I. (2019). Convolution neural network for text mining and natural language processing. *IOP Conf. Ser. Mater. Sci. Eng.* 662:52010. 10.1088/1757-899x/662/5/052010

[B62] Xie Z. Schwartz O. Prasad A. (2018). Decoding of finger trajectory from ECoG using deep learning. *J. Neural Eng.* 15:36009. 10.1088/1741-2552/aa9dbe 29182152

[B63] Yeganegi H. Fathi Y. Erfanian A. (2018). Decoding hind limb kinematics from neuronal activity of the dorsal horn neurons using multiple level learning algorithm. *Sci. Rep.* 8:577. 10.1038/s41598-017-18971-x 29330489 PMC5766487

[B64] Zhang X. Yao L. Wang X. Monaghan J. McAlpine D. Zhang Y. (2021). A survey on deep learning-based non-invasive brain signals: recent advances and new frontiers. *J. Neural Eng.* 18:31002. 10.1088/1741-2552/abc902 33171452

[B65] Zou H. Hastie T. (2005). Regularization and variable selection via the elastic net. *J. R. Stat. Soc. Ser. B* 67 301–320. 10.1111/j.1467-9868.2005.00503.x

